# Vitrectomy for bilateral macular schisis without apparent optic disc anomalies

**DOI:** 10.3205/oc000048

**Published:** 2016-09-13

**Authors:** José Andonegui, José Ramón Maya, Marta Echeverría, Araceli Alcaine

**Affiliations:** 1 Department of Ophthalmology, Complejo Hospitalario de Navarra, Pamplona, Spain; 2 Department of Ophthalmology, The Royal Wolverhampton NHS Trust, Wolverhampton, UK

**Keywords:** macular schisis, optic disc, vitrectomy

## Abstract

A 78-year-old man complained of bilateral visual acuity loss. Optical coherence tomography examination showed bilateral macular schisis with fluid accumulation in the external retinal layers without vitreous traction. Fundus examination and fluorescein angiography were normal in both eyes. Both eyes were treated by phacoemulsification, intraocular lens implantation, and vitrectomy without laser, gas exchange, or retinal fenestration. Slow and progressive fluid resorption and improvement in VA were observed in both eyes.

Macular schisis similar to the one associated with optic disc anomalies is a possibility in patients without apparent disc anomalies. Vitrectomy without laser, gas, or retinal fenestration may be a good therapeutic option even in patients with a PVD preoperatively.

## Introduction

Macular schisis refers to splitting of the central retina usually associated with congenital anomalies of the optic disc including an optic pit, optic nerve coloboma, morning glory anomaly, vitreous traction, veno-occlusive disease, or cystoid macular edema. Spontaneous resolution has been reported in some patients but the majority of cases have a poor prognosis with progressive worsening of visual acuity (VA). We report the case of a patient with bilateral macular schisis without an apparent optic disc anomaly who was treated successfully with bilateral vitrectomy. 

## Case description

**Figure 1:**
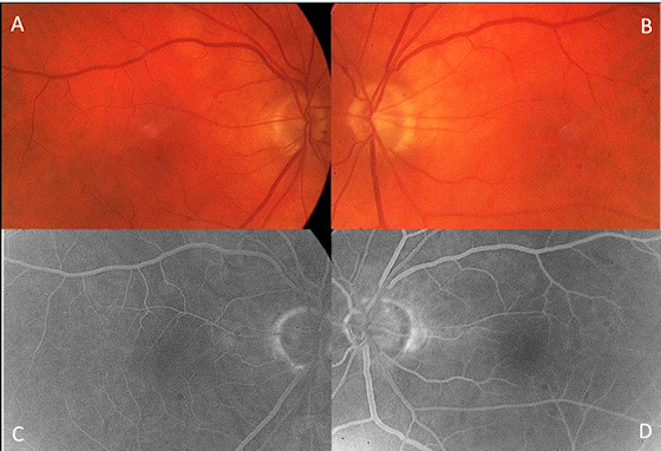
A, Fundus of the right eye. B, Fundus of the left eye. C, Fluorescein angiogram of the right eye. D, Fluorescein angiogram of the left eye.

**Figure 2:**
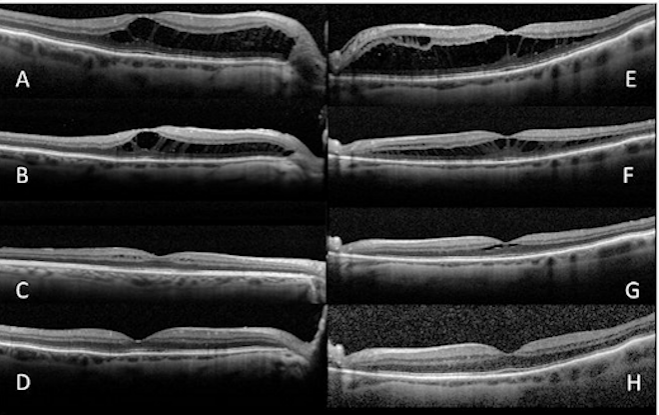
A, Macular schisis of the right eye. B, C, D, Progressive resolution of macular schisis in the right eye. E, Macular schisis of the left eye. F, G, H, Progressive resolution of macular schisis in the left eye.

A 78-year-old man complained of bilateral visual acuity loss over the previous 6 months. The best-corrected VA (BCVA) was 20/40 bilaterally. Anterior segment examination showed moderate bilateral cortical lens opacities. Bilateral fundus evaluations and fluorescein angiography were unremarkable (Figure 1 (Fig. 1)). The retina appeared attached in the posterior pole during fundus examination. Optical coherence tomography showed bilateral macular schisis with abundant fluid accumulation in the external retinal layers emanating from the optic disc (Figure 2 (Fig. 2)). No vitreous traction was observed. Assessment of macular function by perimetry or microperimetry or patient referral to a neurologist was not considered of interest. Both eyes underwent surgery that included phacoemulsification, intraocular lens implantation, and vitrectomy. Laser, gas exchange, or retinal fenestration was not performed as adjuvants intraoperatively. A posterior vitreous detachment (PVD) was detected intraoperatively in both eyes. An interval of 3 months was allowed between both surgeries in each eye. Progressive fluid resorption and improvement in VA were observed bilaterally; 12 months postoperatively the bilateral BCVA was 20/20 and the macular schisis resolved bilaterally (Figure 2 (Fig. 2)). 

## Discussion

Maculopathy associated with optic disc anomalies is an enigmatic disease in which the source of fluid and the pathogenic mechanism remain controversial [1]. The vitreous cavity and the subarachnoid space have been considered the most plausible sources of the fluid. Regarding the pathogenic mechanism, some authors believe that vitreous traction on the peripapillary retina leads to a schisis-like accumulation of fluid, but the exact mechanism is unknown [2]. Another possible mechanism implicates dynamic pressure gradients between the vitreous cavity and the subarachnoid space together with the characteristic anatomic features of cavitary disc anomalies. While the mean intraocular pressure was 16 mmHg, normal intracranial pressure ranges from 5 to 15 mmHg. However, large fluctuations in intracranial pressure have been measured due to factors such as bodily position or venous pressure. According to the dynamic pressure gradients theory, decreases in intracranial pressure result in passage of liquefied vitreous into the subarachnoid space around the optic disc. When the intracranial pressure rises, the fluid is ejected into the vitreous cavity, but small aliquots can migrate into the retina leading to progressive macular schisis contiguous to the optic disc [1].

Few reports have been published on patients with macular schisis without apparent optic disc anomalies and none has reported bilateral involvement or treatments [3], [4], [5]. Another intriguing characteristic of the current patient was the good response to vitrectomy without laser or gas tamponade. Several therapeutic alternatives have been proposed for this entity, but there is little consensus in the literature with respect to the most adequate treatment in these patients. Options available include laser photocoagulation, macular buckling, vitrectomy alone, and vitrectomy associated with laser, gas, or retinal fenestration [1], [2], [6]. Laser photocoagulation can induce visual field defects and recent evidence indicates that it does not aid in anatomical success [7].

Vitrectomy without gas tamponade has shown good results in patients without PVD preoperatively [2]. Supporters of this surgery consider that peripapillary vitreous traction triggers the schisis-like accumulation of fluid. In the current case, complete bilateral fluid resorption was achieved postoperatively despite development of the bilateral PVDs preoperatively. We hypothesized that dynamic pressure gradients caused the macular schisis in our patient. Vitrectomy leads to resolution of macular schisis by altering the fluid dynamics in the vitreous cavity. 

We concluded that macular schisis similar to the one associated with optic disc anomalies is a possibility in patients without apparent disc anomalies. Vitrectomy alone may be a good therapeutic option even in patients with a PVD preoperatively.

## Notes

### Competing interests

The authors declare that they have no competing interests.

### Statement of ethics

The patient signed an informed consent before the surgery.
